# Identification of Conserved and HLA Promiscuous DENV3 T-Cell Epitopes

**DOI:** 10.1371/journal.pntd.0002497

**Published:** 2013-10-10

**Authors:** Eduardo J. M. Nascimento, Robbie B. Mailliard, Asif M. Khan, John Sidney, Alessandro Sette, Nicole Guzman, Michael Paulaitis, Andréa Barbosa de Melo, Marli T. Cordeiro, Laura V. G. Gil, Françoir Lemonnier, Charles Rinaldo, J. Thomas August, Ernesto T. A. Marques

**Affiliations:** 1 Department of Infectious Disease and Microbiology, University of Pittsburgh, Pittsburgh, Pennsylvania, United States of America; 2 Center for Vaccine Research, University of Pittsburgh, Pittsburgh, Pennsylvania, United States of America; 3 Department of Pharmacology and Molecular Sciences, The Johns Hopkins University, Baltimore, Maryland, United States of America; 4 Perdana University Graduate School of Medicine, Serdang, Selangor Darul Ehsan, Malaysia; 5 La Jolla Institute for Allergy and Immunology, La Jolla, California, United States of America; 6 Department of Chemical & Biomolecular Engineering, Ohio State University, Columbus, Ohio, United States of America; 7 Department of Virology and Experimental Therapy, CPqAM/FIOCRUZ, Recife, Pernambuco, Brazil; 8 Institut Pasteur, Unité Immunité Cellulaire Antivirale, Paris, France; University of California, Berkeley, United States of America

## Abstract

Anti-dengue T-cell responses have been implicated in both protection and immunopathology. However, most of the T-cell studies for dengue include few epitopes, with limited knowledge of their inter-serotype variation and the breadth of their human leukocyte antigen (HLA) affinity. In order to expand our knowledge of HLA-restricted dengue epitopes, we screened T-cell responses against 477 overlapping peptides derived from structural and non-structural proteins of the dengue virus serotype 3 (DENV3) by use of HLA class I and II transgenic mice (TgM): A2, A24, B7, DR2, DR3 and DR4. TgM were inoculated with peptides pools and the T-cell immunogenic peptides were identified by ELISPOT. Nine HLA class I and 97 HLA class II novel DENV3 epitopes were identified based on immunogenicity in TgM and their HLA affinity was further confirmed by binding assays analysis. A subset of these epitopes activated memory T-cells from DENV3 immune volunteers and was also capable of priming naïve T-cells, *ex vivo*, from dengue IgG negative individuals. Analysis of inter- and intra-serotype variation of such an epitope (A02-restricted) allowed us to identify altered peptide ligands not only in DENV3 but also in other DENV serotypes. These studies also characterized the HLA promiscuity of 23 HLA class II epitopes bearing highly conserved sequences, six of which could bind to more than 10 different HLA molecules representing a large percentage of the global population. These epitope data are invaluable to investigate the role of T-cells in dengue immunity/pathogenesis and vaccine design.

## Author Summary

Although there is an increased recognition of the role of T-cells in both dengue pathogenesis and protection, comprehensive analysis of T-cell activation during dengue infection is hampered by the small repertoire of known human dengue T-cell epitopes. Although dengue serotype 3 (DENV3) is responsible for numerous outbreaks worldwide, most of the known epitopes are from studies of dengue 2 serotype (DENV2). In this study, we identified novel DENV3 T-cell epitopes in HLA transgenic mice that were confirmed by HLA binding assays. A subset of these epitopes activated memory T-cells from subjects who were dengue IgG positive and primed naïve T-cells from dengue IgG negative individuals. Notably, some of HLA class II epitopes bearing highly conserved regions common to all four dengue serotypes could bind to multiple HLAs. We postulate that these highly conserved and HLA promiscuous T-helper epitopes can be important components of a dengue tetravalent vaccine.

## Introduction

Dengue is a member of the genus *Flavivirus* with a positive sense, single stranded RNA genome of ∼10 kb. The genome encodes for a polyprotein that is co- and post-translationally cleaved into 10 proteins: three structural (capsid, precursor membrane and envelope), which constitute the virus particle; and seven non-structural proteins (NS1, 2a, 2b, 3, 4a, 4b and 5), which are proteases that cleave the viral polyprotein and contribute to the formation of the replication complex [1], [2], [3]. The virus exists in nature as a complex population of four dengue serotypes (DENV1, 2, 3 and 4), consisting of up to 86% homology of amino acid sequences between serotypes [4].

DENV infection, transmitted primarily by the *Aedes aegypti* mosquito, is a major global health problem in tropical and subtropical areas [5]. The typical spectrum of the dengue disease ranges from asymptomatic to a mild form of the disease, dengue fever (DF). However, a small fraction of the patients develops a severe form of the disease, characterized by increased vascular permeability (dengue hemorrhagic fever - DHF) that can lead to hypovolemic shock (dengue shock syndrome, DSS) and even death.

The leading theories underlying DHF immunopathology are based on the observation that sequential infection with different dengue serotypes leads to greater risk of developing a more severe form of the disease. The earliest postulated mechanistic theory proposes that cross-reactive and non-neutralizing antibodies would form immune complexes with the viruses, that can mediate enhanced infection of Fcγ receptor-expressing cells [6], [7].

Several dengue vaccine candidates have been shown to induce memory T-cell response that can confer protection against dengue infection [8], [9]. The importance of protective cytotoxic T lymphocyte (CTL) responses in primary dengue infection has been demonstrated in IFNα/βR knock out mice model [10]. Despite the lack of IFN type I responses in this animal model, immunization with dengue CTL and T-helper (Th) cell epitopes has been shown to contribute towards faster clearance of the virus [10], [11]. However, a number of studies have suggested a possible involvement of cross-reactive HLA class I T-cells epitopes in dengue pathogenesis. Memory T-cell clones generated during a primary infection in response to epitopes from one dengue serotype, would cross-react with epitope variants presented during a subsequent infection with a different dengue serotype, to elicit abnormal responses (cytokine storm) associated with capillary leakage [12].

Despite the increased acknowledgement that T-cells play a role in both the pathology of and protection from dengue infection, a more comprehensive analysis of T-cell activation during dengue infection is hampered by the small repertoire of known dengue T-cell epitopes in humans. Most of the known epitopes are associated with DENV2, and are restricted to a small number of human leucocyte antigens (HLAs) [9], [13], [14], [15], [16], [17], [18], [19]. Reported T-cell epitopes from DENV3, however, are limited and mostly in NS3 protein [15], [16], [18], [20], [21], [22], [23], [24], [25], [26], [27], [28], [29], [30]. The search for DENV3 T-cell epitopes has been motivated by the association of DENV3 with major outbreaks in the Americas and Southeast Asia, infecting adults and children and causing a wide spectrum of disease severity [31], [32], [33], [34], [35], [36], [37], [38]. Thus, the search for DENV3 T-cell epitopes is necessary.

We previously showed that T-cell responses elicited either by attenuated yellow fever vaccine (17DD) or peptide immunization were similar in terms of epitope repertoire and immune dominance [39]. We also demonstrated a correlation between the strength of binding to HLA class I and epitope immunogenicity [39], [40]. Based on these studies, we devised an optimized strategy for identification and characterization of DENV3 T-cell epitopes by use of overlapping peptide libraries which were constructed based on protein sequences of DENV3 isolates of our human cohort [37] followed by *in silico* and biochemical characterization of the immunogenic peptides. This strategy was applied to HLA transgenic mice, an effective animal model to use for identifying potential epitopes recognized by human T-cells. The repertoire of epitopes identified in these animal models has been correlated with those identified in humans [41], [42], [43], and thus, the strategy was proven to be an effective platform for epitope discovery [44].

Recently, IFN α/βR KO mice were backcrossed with different HLA transgenic mice [17] and used to discover HLA-A02-restricted T-cell epitopes upon infection with a mice-adapted DENV2 S221 strain. In the current study, however, we used a mouse model with functional IFN α/βR and identified T-cell epitopes of DENV3 Envelope, NS1, NS3 and NS5 proteins by use of transgenic mice expressing HLA class I (A2, A24 and B7) and II (DR2, DR3 and DR4) molecules. The affinity of the epitopes to their specific HLAs was confirmed by binding assays. A subset of the epitopes identified the presence of activated memory T-cells from subjects naturally infected with DENV3, and also effectively primed naïve T-cell clones from dengue IgG negative individuals (dengue naïve). Additionally, analysis of intra- and inter-serotype variants was carried out for one HLA class I epitope for identification of possible altered epitope ligands in other DENV serotypes. This could facilitate the identification of epitope variants that could cause aberrant memory T-cell activation.

An effective dengue vaccine needs to provide broad population coverage and induce immune response against majority variants of the four dengue serotypes. Therefore it would be desirable for a dengue vaccine to target the T-cell responses against conserved amino acid sequences that can be presented by multiple HLAs. In our previously reported preliminary study, we showed that epitopes containing highly conserved dengue sequences (pan-dengue sequences; defined as being present in each of the four dengue serotypes with an incidence of 80% or more [45]) are immunogenic in HLA TgM. Herein, we showed that such sequences can also be quite promiscuous, binding as many as 14 different HLA molecules. The results suggest that T-cell epitopes containing highly conserved in each of the four dengue serotypes could be immunogenic in a large percentage of the global population and, thus, potentially useful for further exploration as a vaccine target against DENV.

## Materials and Methods

### Ethics statement

This study was performed in strict accordance to the recommendations in the Guide for the Care and Use of Laboratory Animals of the National Institutes of Health. The TgM protocol was approved by The Johns Hopkins University (JHU) Institutional Animal Care & Use Committee (MOO7M78). Adult subjects with history of dengue infection analyzed in this study were recruited from a cohort of suspected dengue cases established in Recife, Pernambuco, Brazil and described by Cordeiro *et al.* [37]. All patients provided their written informed consent to participate and this study was reviewed and approved by the ethics committee of the Brazilian Ministry of Health (CONEP: 4909; Process n° 25000.119007/2002-03; CEP: 68/02). In addition, the Johns Hopkins University Institutional Review Board reviewed and approved the study (protocol JHM-IRB-3: 03-08-27-01).

### Peptide library

Synthetic peptides covering the full-length Envelope (Env; 95 peptides), NS1 (75), NS3 (150), and NS5 (156) protein sequences of DENV3 Philippines/H87/1956 isolate (UniProtKB/Swiss-Prot Accession: P27915; GenPept: AAA99437) were obtained for the study (Supplementary Table S1). The Env, NS1 and NS3 peptides were 15-mers, overlapping by 10–11 amino acids (aa), and synthesized by Schafer-N (Denmark), while the NS5 peptides were 13- to 17-mers, overlapping by 11 to 13 aa, and obtained from BEI Resources, NIH (Supplementary Table S1). All the 9-mer peptides used for HLA class I minimal epitope discovery and analysis of the variants of DENV NS3_399–407_ were purchased from Genscript Corporation. All peptides were dissolved in 10% (v/v) dimethyl sulfoxide (DMSO; Sigma) at 2 mg/mL, aliquoted and stored at −20°C until use.

### HLA transgenic mice (TgM)

Murine H-2 class II-deficient, HLA-A02 (A*0201) [46], HLA-B07 (B*0702) [47], HLA-A24 (A*2402) (Lemonnier *et al.* unpublished), HLA-DR2 (DRA1*0101/I-Eα; DRB1*1501/I-Eβ) [48], HLA-DR3 (DRA1*0101; -DRB1*0301) [49], [50], and HLA-DR4 (HLA-DRA1*0101; -DRB1*0401) [51], animals were bred and maintained in the Johns Hopkins University School of Medicine Animal Facility. Specific pathogen-free colonies were maintained in a helicobacter-negative mice facility. HLA expression of the experimental transgenic mice was evaluated by flow cytometry (data not shown).

### Immunizations protocol

Ten to 15 mice were injected twice, two weeks apart, with a pool of peptides (1 µg/peptide) emulsed in Titermax gold (Titermax) or only Titermax gold (negative control) according to the manufacturer directions. The immunization was performed through subcutaneous route.

### Tissue processing and splenocyte isolation

Two weeks after the last immunization, the mice were sacrificed and their spleens were aseptically removed and placed into a disposable sterile petri dish containing 5 mL of RPMI media, followed by manual dissociation and grinding (pressing tissue against a sterile 70 mm cell strainer). After washing the strainer with 15 mL of RPMI media, the splenocytes were transferred into a 50 mL conical tube and centrifuged 500×g for 5 minutes at room temperature (RT). Red blood cells with the splenocytes were lysed by treating the pellets with ammonium-chloride-potassium (ACK) lysing buffer (3 mL per spleen) for 3 minutes at RT. The splenocytes were then washed by addition of 20 mL of RPMI media, centrifuged at 500×g for 5 minutes at 4°C, resuspended, strained and counted using a Vi-Cell analyzer (Beckman Dickson). The isolated splenocytes were depleted of murine CD4 (HLA class I TgM) or CD8 (HLA class II TgM) positive cells by the use of antibody-coated microbeads and LD columns manufactured by Miltenyi Biotec following the instructions of the manufacturer.

### Mapping of T-cell epitopes using HLA transgenic mice

**Figure 1:**
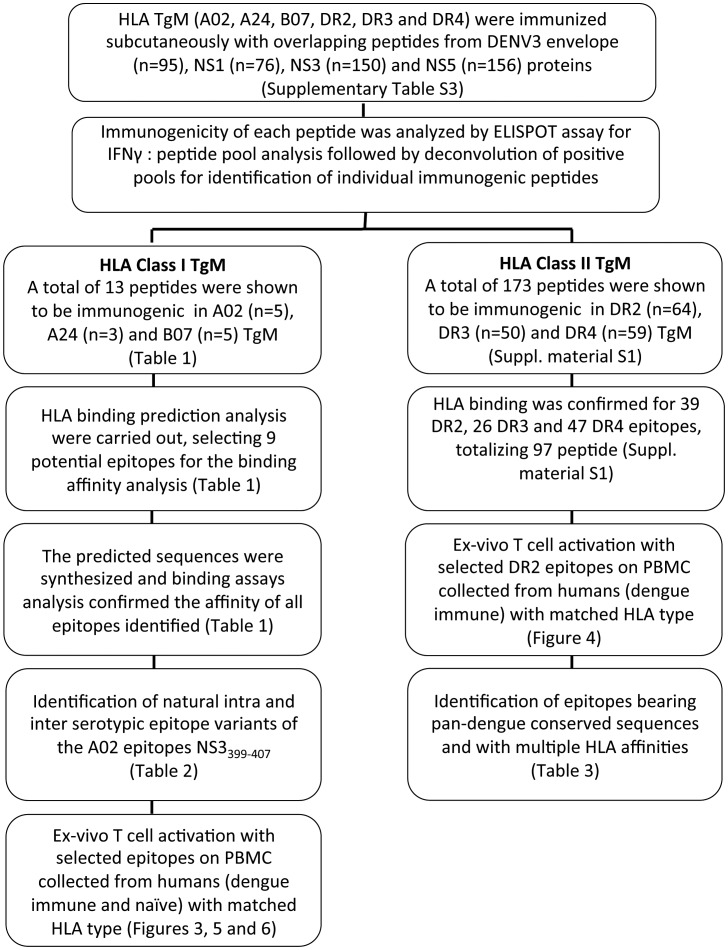
Flowchart showing the high-throughput epitope mapping strategy carried out in this study.

The cells from immunized and control mice were screened by IFN-γ ELISPOT assays for memory T-cell response against either the pooled or individual peptides used in the immunization peptide pool. The strategy for epitope mapping, as depicted on Figure 1, included a first round of screening of overlapping peptides arrayed in different matrix pools (Envelope, 10×10; NS1, 9×9; NS3 and NS5, 13×12), whereby each peptide is present in two different pools [52]. This was followed by a deconvolution of the positive pools. The peptide concentration in both of these steps was 10 µg/mL. Lastly, the functional avidity of the immunogenic peptides was assessed by measuring T-cell response against the peptides at different concentrations (10 µg/mL, 1 µg/mL and 0.1 µg/mL) as previously reported [53].

### HLA typing

Low resolution HLA typing for HLA-A, HLA-B, HLA-Cw, HLA-DR, and HLA-DQ loci was performed using Sequence Specific Primers amplification methods as described elsewhere [54].

### Plaque reduction neutralization test

The primary virus strains DENV-1 (PE/97-42735), DENV-2 (PE/95-3808), DENV-3 (PE/02-95016) and DENV-4 (IEC) isolated in Brazil were expanded on African green monkey kidney cells and used in a standard plaque reduction neutralization test with the same cell line and heat-inactivated patient sera as described [55]. Serum samples were used at two-fold dilutions ranging from 1/20 to 1/2560. The 50% end-point dilution of each serum, corresponding to the dilution at which 50% of the wells were completely protected from infection, was determined according to standard methods. Fifty percent plaque neutralization titers (PRNT50) were calculated as the highest dilution of Ab reducing virus plaques by 50%.

### Human dengue cohort

Peripheral blood samples were obtained from 5 subjects enrolled in this cohort who had history of dengue infection with different virus serotypes, including DENV3, for at least a year, prior to the blood collection. Demographic, dengue serotype-specific immunity status and HLA typing information are shown in supplementary Tables S1 and S2.

### Preparation of human PBMC

Blood from the subjects was collected in heparinized tubes (BD Biosciences) 1 to 3 years year after onset of symptoms. PBMCs were isolated by gradient density using Ficoll-Paque Plus according to the instructions of the manufacturer (GE Healthcare). The cells were then washed once with PBS (Phosphate Buffer Saline) pH 7.2 and the red cells were lysed with ACK Lysing buffer (Biosource International, Inc) for 3 minutes at room temperature (RT). The cells were then centrifuged at 530×g for 5 minutes at 4°C and re-suspended, strained and counted by use of cell analyzer Vi-Cell XR (Beckman Coulter).

PBMC from HLA-A02 or HLA-B07 positive volunteers were used to assess the immunogenicity of the HLA-class I epitopes while HLA-DR2 positive donors were used to assess the immunogenicity of HLA-class II epitopes. The CD4^+^ T-cells and CD8^+^ T-cells were depleted from the HLA-A02/B07 positive donors and the HLA-DR2^+^ donors respectively. Cell depletion was carried out by magnetic bead cell isolation using Miltenyi CD4^+^ and CD8^+^ microbeads and LD columns (Miltenyi Biotec) according to the manufacturer manual. Following the cell depletion step, the remaining cells were suspended at 2.5×10^6^ cell/mL and cultured at 37°C, 5% CO2 in RPMI media containing 8% (v/v) T-cell growth factor (TCGF), 5% (v/v) autologous plasma. T-cells were activated with individual peptides at 10 µg/mL. On day 4, the cell cultures were replenished with TCGF and autologous plasma. On day 8, the cells were harvested and washed with serum-free media (Gibco) to prepare for ELISPOT analysis as described below.

### ELISPOT assays for IFN-γ

ELISPOT assays for IFN-γ detection were performed using the IFN-γ ELISPOT set from BD Biosciences, according to the manufacturer's instructions. Briefly, the ELISPOT plate (96-well) was coated with anti-mouse IFN-γ at 5 µg/mL and incubated at 4°C overnight. The plate was blocked with RPMI-1640 containing 10% heat-inactivated fetal bovine serum (HyClone), 1% L-Glutamine (Gibco), 1% penicillin/streptomycin (Gibco) for 2 h at RT. Peptide pools or individual peptides were then plated in duplicate. The cells were then added at a range of 5 to 10×10^5^ cells/well. Concanavalin A (Sigma), at 2.5 µg/mL, was used as a positive control while media was used as a negative control (background). After an 18 h incubation at 37°C and 5% CO2, the plate was washed and incubated with biotinylated anti-mouse IFN-γ at 2 µg/mL for 2 h at RT. Streptavidin-HRP 100-fold diluted was then added and incubated for 1 h at RT. The plate was washed and reactions were developed with 3-amino-9-ethylcarbazole (AEC) substrate (BD Biosciences Pharmingen). Spot development was stopped after 30 min incubation by washing the plate with distilled water. The plate was dried at RT and the spots were counted with the Immunospot Series 3B Analyser ELISPOT reader (Cellular Technologies Ltd) using the program Immunospot software version 3.0 (Cellular Technologies Ltd). The spot average was normalized and expressed as the number of spot-forming cells (SFC) per 1 million cells.

ELISPOT assays were also used to assess human T-cell responses using a protocol similar to the above using a human IFN-γ ELISPOT kit (BD Biosciences); phorbol 12-myristate 13-acetate (PMA; Sigma) at 250 ng/mL and ionomycin (Sigma) at 250 ng/mL were used as a positive assay control. Peptide pools and individual peptides were considered positive in the ELISPOT assay when all three criteria described bellow were met:

- *mean number of spots (peptide pool or individual peptides) minus 2 standard deviation (SD) was greater than the mean number of spots (background);*
- *mean number of spots (peptide pool or individual peptides) was greater than the mean number of spots (background) plus 2SD;*
- *mean number of spots (peptide pool or individual peptides) minus mean number of spots (background) was greater than 10 spots per million cells.*

These criteria were used consistently throughout this study.

### Generation of human DCs and induction of primary CTL responses

Monocytes were isolated from peripheral blood of healthy donors (Central Blood Bank of Pittsburgh, USA) and cultured for 5–7 days in 24-well plates at 5×105 cells per well in the presence of GM-CSF and IL-4 (both 1000 IU/ml). The immature DCs were then exposed to the following combination of activation factors for 48 h: rhIL-1β (25 ng/ml), rhTNFα (50 ng/ml), rhIFNα (1000 IU), Poly IC (20 µg/ml) and IFNγ (1000 IU/ml). As previously described [56], high IL-12 p70 producing type-1 polarized DC (αDC1) were induced

CTL were generated using a protocol similar to what we previously described [56]. Briefly, CD8+ T-cells were isolated from the peripheral blood of dengue virus naïve (dengue IgG negative) HLA-A2+ donors by negative selection using the EasySep system (Stem Cell Technologies). The T-cells were plated at 7.5×10^5^cells/well in 48 well plates (Falcon, BD Labware) and sensitized with 9-mer peptide-pulsed autologous αDC1 (7.5e4 cells/well). To mimic their interaction with CD40L-expressing CD4+ T-helper cells, as a surrogate we added to the cultures γ-irradiated (3000 Rad) J558-CD40L cells (5e4 cells/well) (provided as a gift from Dr. P Lane, University of Birmingham, Birmingham, U.K.). From day 4 onwards, rhIL-2 (50 units/ml), rhIL-7 (10 ng/ml), and rhIL-15 (100 IU/ml) were added to the cultures every 4 days upon media replacement. The cultures were provided one round of in vitro sensitization at day 14 by adding relevant peptide-pulsed γ−irradiated (3000 Rad) A2+ T2 cells (provided by Dr. W Storkus, University of Pittsburgh) at target cells to T-cell responder ratio of 1∶5. On day 20, the T-cells were screened for the presence of antigen specific CTL by IFN-γ ELISPOT assay described previously. Long term CTL lines from ELISPOT positive cultures were established and maintained under the described culture conditions with IL-2 concentration being increased to 500 IU/ml. The cultures were re-sensitized with peptide pulsed γ−irradiated T2 cells every 10 days and used periodically during the study.

### Prediction of HLA class I epitopes and determination of HLA class II core region interaction

Prediction of HLA class I T-cell epitopes for HLA-A*0201, HLA-A*2402 and HLA-B*0702 molecules was performed by use of the ANN (artificial neural network) algorithm based prediction tool at the Immune Epitope Database (IEDB) and Analysis Resource (http://tools.immuneepitope.org/analyze/html/mhc_binding.html). The peptide sequences (13-mer to 17-mer) were input to the prediction server and prediction was carried out for all possible lengths (8-mer to 11-mer). Peptide sequences predicted with IC50 below 500 nM were considered for further analysis.

For determination of HLA class II core region interaction within the peptides bearing pan-dengue conserved sequences, stabilization matrix method (SMM), artificial neural network (NN) and Sturniolo alignment algorithms [57], [58], [59] were used. The peptide sequences were input to the IEDB server for identification of HLA class II epitopes under the IEDB recommended prediction method. Then, the 9-mer sequence of align core (core region interaction) for the lowest predicted IC50 was annotated for each prediction method.

### HLA purification and binding assays

Purification of HLA class I (A*2402 and B*0702) and HLA class II molecules (DRB1*0101, DRB1*0301, DRB1*0401, DRB1*0404, DRB1*0405, DRB1*0701, DRB1*0802, DRB1*0901, DRB1*1101, DRB1*1302, DRB1*1501, DRB3*0101, DRB4*0101 and DRB5*0101) by affinity chromatography and the quantification of peptide binding based on competitive inhibition assay against binding of a high affinity radiolabeled standard peptide were performed as detailed elsewhere [60], [61], [62], [63], [64]. Briefly, EBV transformed homozygous cell lines were used as sources of HLA molecules. A high affinity radiolabeled peptide (0.1–1 nM) was co-incubated at room temperature or 37C with purified HLA in the presence of a cocktail of protease inhibitors. Following a two-day incubation, HLA bound radioactivity was determined by capturing HLA/peptide complexes on Ab coated Lumitrac 600 plates (Greiner Bio-one, Frickenhausen, Germany), and measuring bound cpm using the TopCount (Packard Instrument Co., Meriden, CT) microscintillation counter. The concentration of peptide yielding 50% inhibition of the binding of the radiolabeled peptide was calculated. Under the conditions utilized, where [label]<[HLA] and IC50≥[HLA], the measured IC50 values are reasonable approximations of the true Kd values. Each competitor peptide was tested at six different concentrations covering a 100,000-fold range, and in three or more independent experiments. As a positive control, the unlabeled version of the radiolabeled probe was also tested in each experiment.

### Dengue competitive binding assay using HLA-A2:Ig fusion protein

Binding assay for HLA-A*0201 was performed using HLA-A2:Ig fusion protein. The labeled peptide (ALMDKVLKV) was commercially synthesized by GenScript Corporation as a reference peptide for the binding assay. A fluorescein moiety was introduced into position P8 of the designed peptide (ALMDKVLKV). This chemical modification has been previously shown to not affect the peptide's affinity for the HLA*A2 binding pocket [65]. The lyophilized labeled peptide was diluted in 100% DMF (Dimethylformamide) to a final concentration of 10 mM. Subsequent dilutions were carried out using 0.5 mg/ml bovine-γ-globulin (BGG) in 1×PBS.

Soluble HLA-A2:Ig dimer complexes were obtained from BD Biosciences. This fusion protein consists of two extracellular portions of the major histocompatibility complex (MHC) class I HLA-A2 domains fused to the variable regions of a mouse IgG1 antibody. Additional β2-microglobulin (Fitzgerald Industries) was added to the binding assays to retain the fusion protein in a functional state. Fluorescence polarization was measured by a SpectraMax M5 reader (Molecular Devices, Sunnyvale CA) using an excitation wavelength of 485 nM and an emission wavelength of 530 nM. The competitive binding assays were carried out at room temperature for an incubation period of 3 days.

Both labeled and unlabeled peptides were added to the reaction mixture before the HLA-A2:Ig fusion protein, ensuring that both peptides were presented simultaneously to the HLA molecule. To avoid peptide photobleaching, the reaction mixture was incubated within aluminum foil covered microcentrifuge tubes. After the 3 days incubation period, 20 µl of the reaction mixture was loaded into individual wells of a black, non-binding surface (NBS) 384 well plate (Corning). Controls included only buffer (blank), protein and pFITC. Each experiment was performed in quadruplicate and reported as the mean with standard deviation. The reported IC50 values were obtained by fitting the mean of 13 data points to a four-parameter logistic equation:
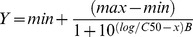
where, max indicates the upper plateau of the curve; min, the lower plateau and B, is the slope factor, which describes the steepness of the curve transition.

### Diversity analysis HLA-A*0201-restricted DENV3 NS3_399–407_ epitope and variants

NS3 protein sequences of each DENV serotype (696 for DENV1, 681 DENV2, 585 DENV3, and 71 DENV4; as of Feb. 2009) were collected from the NCBI Entrez Protein Database and aligned, following the method in Khan *et al.*, (2008) [45]. The region of each serotype alignment corresponding to the DENV3 NS3_399–407_ epitope sequence (KLNDWDFVV) was extracted and analysed for all the peptides that were variant/different to the epitope sequence by at least one amino acid difference. The incidence (% occurrence) of the individual variants in each DENV serotype NS3 alignment was determined. Inter-serotype variant peptides (DENV1, 2 and 4) with an incidence of 5% or more and predicted to be a potential epitope of HLA-A*0201 were synthesized and their binding affinity and immunogenicity assayed.

## Results

### Epitope identification in HLA transgenic mice

The immunogenicity of each of the 477 peptides from DENV3 envelope (Env), NS1, NS3 and NS5 proteins (Supplementary Table S3) were tested in six HLA transgenic mice (TgM) strains (A02, A24, B07, DR2, DR3 and DR4) using IFN-γ ELISPOT as the initial screening readout (Figure 1). A total of 13 novel peptides were shown to be immunogenic in HLA class I transgenic mice (TgM) [A02 (n = 5), A24 (n = 3) and B07 (n = 5)], six of which were located in Env, three in NS3 and four in NS5 (Table 1). No HLA class I epitope was found in NS1 protein. A02 and B07 epitopes were presented in Env, NS3 and NS5, whereas A24 epitopes were present only in Env. In contrast to HLA class I, a total of 173 peptides were shown to be immunogenic in HLA class II TgM [DR2 (n = 64), DR3 (n = 50) and DR4 (n = 59) (Supplentary material S3)], 36 of which were located in Env, 31 in NS1, 48 in NS3, and 58 in NS5 (Supplementary Table S4).

**Table 1:** Identification of DENV3 HLA class I (A02, B07 and A24) T-cell epitopes using HLA transgenic mice, epitope prediction and binding assays.

Transgenic Mice	Peptide	Sequence	SFC±SD (Functional avidity in µg/mL)	Predicted sequence	Binding affinity (nM)
A02	Env_106–120_	GLFGKGSLVTCAKFQ	11±0 (10)	GLFGKGSLV	2.9
	NS3_393–407_	TEYQKTKLNDWDFVV	20±5 (0.1)	KLNDWDFVV	110
	NS5_227–243_	IVSSVNMVSRLLLNRFT	44±11 (10)	None	-
	NS5_312–328_	ATGSASSMINGVVKLLT	81±8 (1)	SMINGVVKL	281
	NS5_318–334_	SMINGVVKLLTKPWDVV	89±22 (0.1)	SMINGVVKL KLLTKPWDV	281619
A24	Env_296–310_	SYAMCTNTFVLKKEV	148±9 (1)	SYAMCTNTF	14
	Env_441–455_	AYTALFSGVSWVMKI	18±4 (10)	None	-
	Env_466–480_	LNSKNTSMSFSCIAI	31±0 (1)	None	-
B07	Env_221–235_	GATTETPTWNRKELL	192±2 (10)	TPTWNRKEL	2.8
	Env_226–240_	TPTWNRKELLVTFKN	456±7 (0.1)		
	NS3_589–603_	KKKLRPRWLDARTYS	115±5 (1)	RPRWLDART	2.1
	NS3_593–607_	RPRWLDARTYSDPLA	139±3 (1)		
	NS5_382–398_	RTLGRNKRPRLCTREEF	501±19 (0.1)	RPRLCTREEF	7.1

### Binding assays

Affinity of the immunogenic peptides to the respective HLA of the TgM was confirmed by use of HLA-binding assay. HLA class I binding groove fits peptides between 8 to 12 amino acid (aa) long (typically 9 aa). Hence, *in silico* analysis was first performed to identify 9-mer(s) within the immunogenic peptides (15-mers to 17-mers) that were potential binders of the respective HLA of the TgM. Nine out of the 13 immunogenic peptides encompassed binding motifs for the HLA they were immunogenic in the animal studies and, thus, they were selected for peptide synthesis and binding assay analysis, which further confirmed the affinity of all tested peptides (Table 1).

The open topology of the HLA class II groove, fitting longer peptides than class I, allowed direct use of the immunogenic peptides (15-mer to 17-mer) for the binding assay, without the preliminary *in silico* binding prediction. Binding assay was performed against the HLA of three class II TgM [DR2 (DRB1*1501), DR3 (DRB1*0301) and DR4 (DRB1*0401)], as well as eleven additional HLA class II molecules (see methods) for the analysis of epitope promiscuity. HLA binding affinity was confirmed for 39 DR2-, 26 DR3- and 47 DR4-specific epitopes, representing 65%, 53% and 84% of the total positive peptides of DR2, DR3 and DR4 TgM respectively (Supplementary Table S5). Furthermore, many of the peptides tested showed affinity to multiple HLA molecules (supplementary material S1).

### HLA class II epitope promiscuity based on immunogenicity and binding affinity

**Figure 2:**
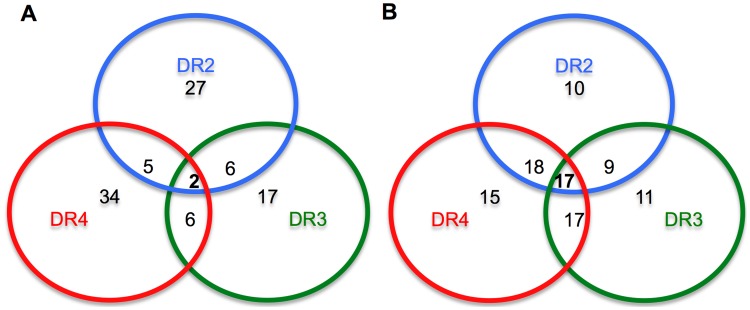
HLA affinity overview among the epitopes identified. (A) Profile based exclusively on immunogenicity in HLA transgenic mice; (B) Profile based exclusively on binding assay analysis.

The number of peptides that elicited T-cell responses in two HLA class II TgMs ranged from 5 to 6 peptides, whereas only 2 peptides were immunogenic in all three HLAs tested in TgM (Figure 2A). The majority of the peptides were positive in only one TgM strain analyzed. However, the binding assay analysis suggests that these peptides are more promiscuous. The majority of the class II peptides had binding affinity to more than one HLA. The number of peptides that had affinity to DR2/DR3, DR2/DR4, DR3/DR4 and DR2/DR3/DR4 were 9, 18, 6 and 17, respectively (Figure 2B).

### Imunogenicity of the epitopes in human: Epitope processing and priming analysis in human subjects

**Figure 3:**
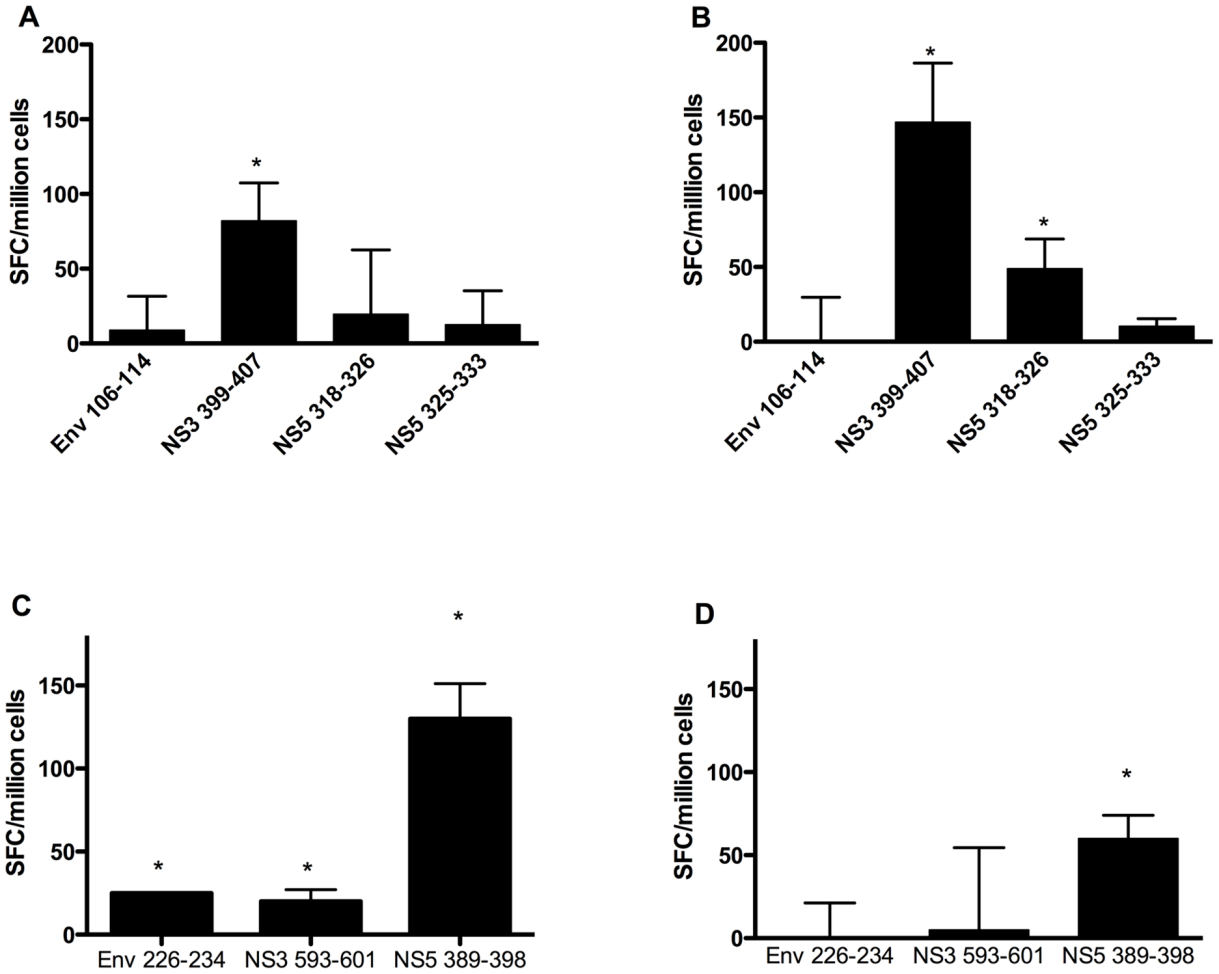
T-cell responses triggered by HLA-A*0201 and HLA-B*0702 epitopes in subjects with history of DENV3 infection. CD4 depleted PBMCs were cultured for a week in presence of peptide pool containing either A*0201 or B*0702 epitopes. The cells were then harvested, washed and ELISPOT assay for IFN-γ detection was performed. (A) and (B) represent the T-cell responses of two different HLA-A*02 positive subjects, whereas (C) and (D) represent T-cell responses of two different HLA-B*07 positive individuals.

**Figure 4:**
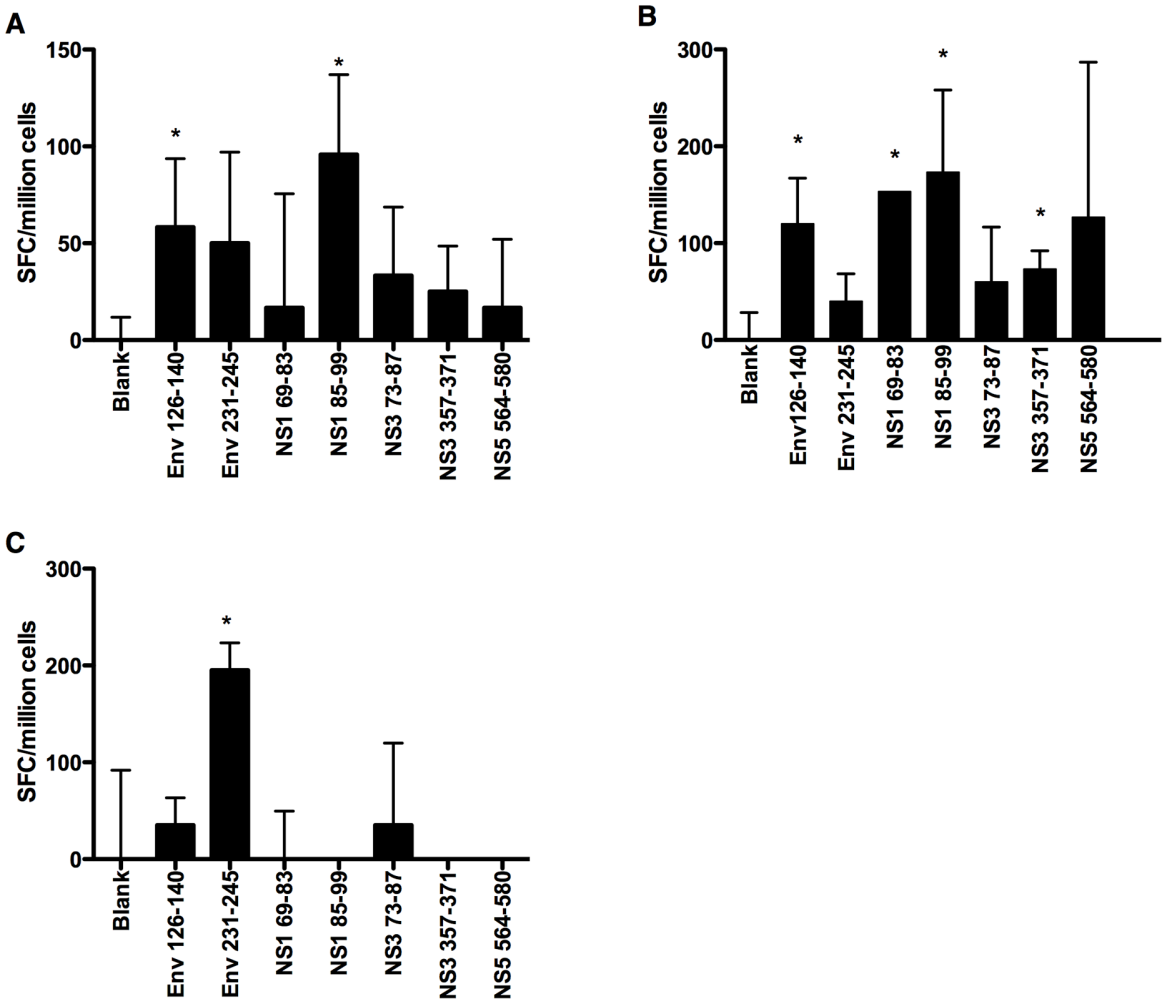
T-cell responses triggered by HLA-DR02 epitopes in subjects with history of DENV3 infection. CD8 depleted PBMCs were cultured for a week with individual peptides. The cells were then harvested and tested for T-cell activation using ELISPOT for IFN-γ as read out. (A), (B) and (C) represent the T-cell responses of three DR02 positive subjects.

TgM and binding affinity assays are useful tools for determination of HLA affinity of an epitope. However, experimental validation of the T-cell epitopes in humans is necessary for accurate interpretation of results. Hence, a subset of the T-cell epitopes, identified herein by use of TgM with HLA affinity confirmed by binding assays, was selected for immunogenicity study in humans by use of PBMC collected from subjects immune to DENV3 (Supplementary Tables S1 and S2). In addition, DR2 epitopes were selected based on their functional avidity, defined as the ability to activate CD4 T-cells at concentrations below 0.1 µg/mL and high affinity to HLA-DR2 molecule (IC50 below 20 nM). This additional criterion was applied in order to reduce the number of DR2 peptides to test and also to select only the peptides with increased likelihood to induce T-cell responses. PBMCs from A02 (n = 2), B07 (n = 2) and DR2 (n = 3) positive subjects were either CD4 depleted (A02 and B07) or CD8 depleted (DR2) and cultured for a week with a peptide pool containing the HLA-matched epitopes, followed by analysis of T-cell activation by use of ELISPOT to detect IFN-γ secretion. Among the A02 positive subjects tested, consistent response against peptide NS3_399–407_ was observed in all subjects, whereas only one of the two responded against the peptide NS5_318–326_ (Figure 3B). No T-cell response was detected against the peptides Env_106–114_ and NS5_325–333_ (Figure 3A & 3B). All subjects analyzed for B07 epitopes elicited T-cell response against the peptide NS5_389–398_ (Figure 3C & 3D). However, T-cell response against Env_226–234_ and NS3_593–601_ was observed only in one of the two subjects analyzed (Figure 3C). The peptides Env_126–140_ and NS1_85–99_ reproducibly activated memory T-cells on all the subjects analyzed for DR2 epitopes (Figure 4). On the other hand, the peptides Env_231–245_, NS1_69–83_ and NS3_357–371_ elicited T-cell response in one of the volunteers while the remaining peptides did not elicit any memory T-cell response (Figure 4). Therefore, majority of the HLA class I and II T-cell epitopes analyzed activated memory T-cell response in at least one individual that had experienced DENV3 infection in the past. This suggests that the epitopes are naturally processed and presented to T-cells during DENV3 infection.

**Figure 5:**
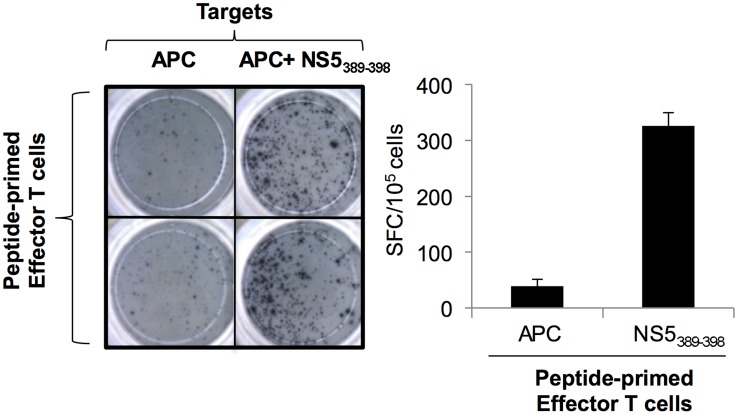
ELISPOT data showing T-cell activation of HLA-B07 positive in dengue IgG negative individuals using dendritic cells pulsed with DENV3 NS5_389–398_ epitope.

**Figure 6:**
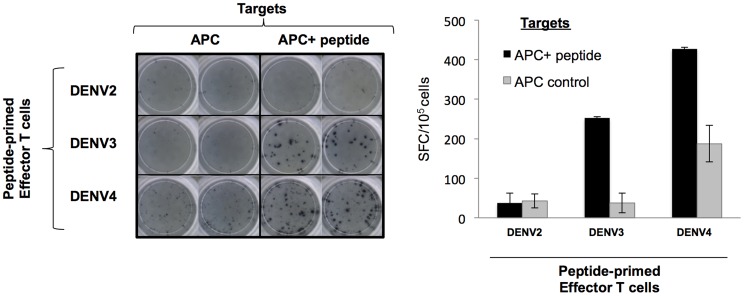
ELISPOT data showing T-cell activation of HLA-A02 positive in dengue IgG negative individuals using dendritic cells pulsed with NS3_399–407_ epitope on homologous region of DENV2, DENV3 and DENV4 viruses.

In addition to showing that the epitopes identified herein are recognized by human memory T-cells, we tested if a subset of the epitopes identified herein could prime naïve T-cells for relevance in epitope-based vaccine design. CD14 positive monocytes were isolated from PBMC harvested from blood collected from healthy donors that were known to be IgG negative for dengue. Monocytes isolated from either HLA-A02 or HLA-B07 positive individuals were differentiated into mature dendritic cells and pulsed with peptide pool containing either A*0201 or B*0702 epitopes, respectively, and co-cultured with autologous lymphocytes responders. After 20 days, the *in vitro*-sensitized T-cells were harvested and reactivity was assessed by IFN-γ ELISPOT using target cells (peptide-pulsed autologous monocytes) expressing either HLA-A*0201 and HLA-B*0702 molecules pulsed with individual peptides. Among the epitopes analyzed, the peptide DENV3 NS5_389–398_ (B*0702-restricted) and NS3_399–407_ (HLA-A*0201-restricted) reproducibly primed naïve T-cells (Figures 5 and 6 respectively). Thus, a selected repertoire of epitopes identified using HLA TgM did not only trigger memory T-cell response in individuals with history of dengue infection, but also primed T-cell from dengue naïve subjects.

### Analysis of variants of DENV3 NS3_399–407_ in other DENV serotypes

Altered peptide ligand is thought to be a mechanism driving memory T-cells toward an aberrant cytokine response associated with disease severity. Intra- and inter-serotype variant analysis of the A02-restricted epitope (DENV3 NS3_399–407_ - KLNDWDFVV) were performed to assess the potential of the peptides to act as altered peptide ligands. The DENV3 NS3_399–407_ epitope had an incidence of 63% among all analyzed DENV3 sequences collected from the NCBI Entrez Protein Database (see Methods section). The remaining ∼37% of the sequences constituted four intra-serotype variants of the epitope (KLNDWDFVV) with only one peptide of incidence more than 1% ( RLNDWDFVV; ∼36%). In contrast, there were 11 inter-serotype variants, but only four (DENV1: KNNDWDYVV; DENV2: RTNDWDFVV and RANDWDFVV; DENV4: KLTDWDFVV) had an incidence of about 5% or more among all analyzed DENV sequences of each serotype (Table 2). Subsequently, HLA binding prediction analysis was performed on the inter-serotype variants to assess the effect of one or two amino acid substitutions to HLA binding. Variants predicted to retain the HLA affinity were synthesized and tested in binding assay experiments. Prediction analysis indicated that only the DENV2 and DENV4 variants retained the affinity to HLA-A*0201, which was experimentally confirmed (Table 2). Further, the DENV2 and DENV4 variants were tested for their ability to prime naïve T-cells was assessed as described in the Methods. DENV2 variant failed to prime naïve T-cells, but DENV4 variant could reproducibly trigger naïve T-cell response *in vitro* (Figure 6) and, thus, it could potentially be an altered peptide ligand to the DENV3 epitope KLNDWDFVV.

**Table 2:** Inter-serotype variants and corresponding incidence among reported sequences in NCBI Entrez database, with binding affinity data to HLA-A*0201 molecule.

Peptide	Sequence	Inter-serotype variant incidence (%)	IC50 (nM)
DENV3	KLNDWDFVV	-	110
DENV1	KNNDWDYVV	46	ND
DENV2 (1)	RTNDWDFVV	26	167
DENV2 (2)	RANDWDFVV	20	ND
DENV4	KLTDWDFVV	5	61

The underlined sequences show the amino acid changes in relation to DENV3 sequence. ND: non-determined.

### Conservation and variability of the epitopes

**Figure 7:**
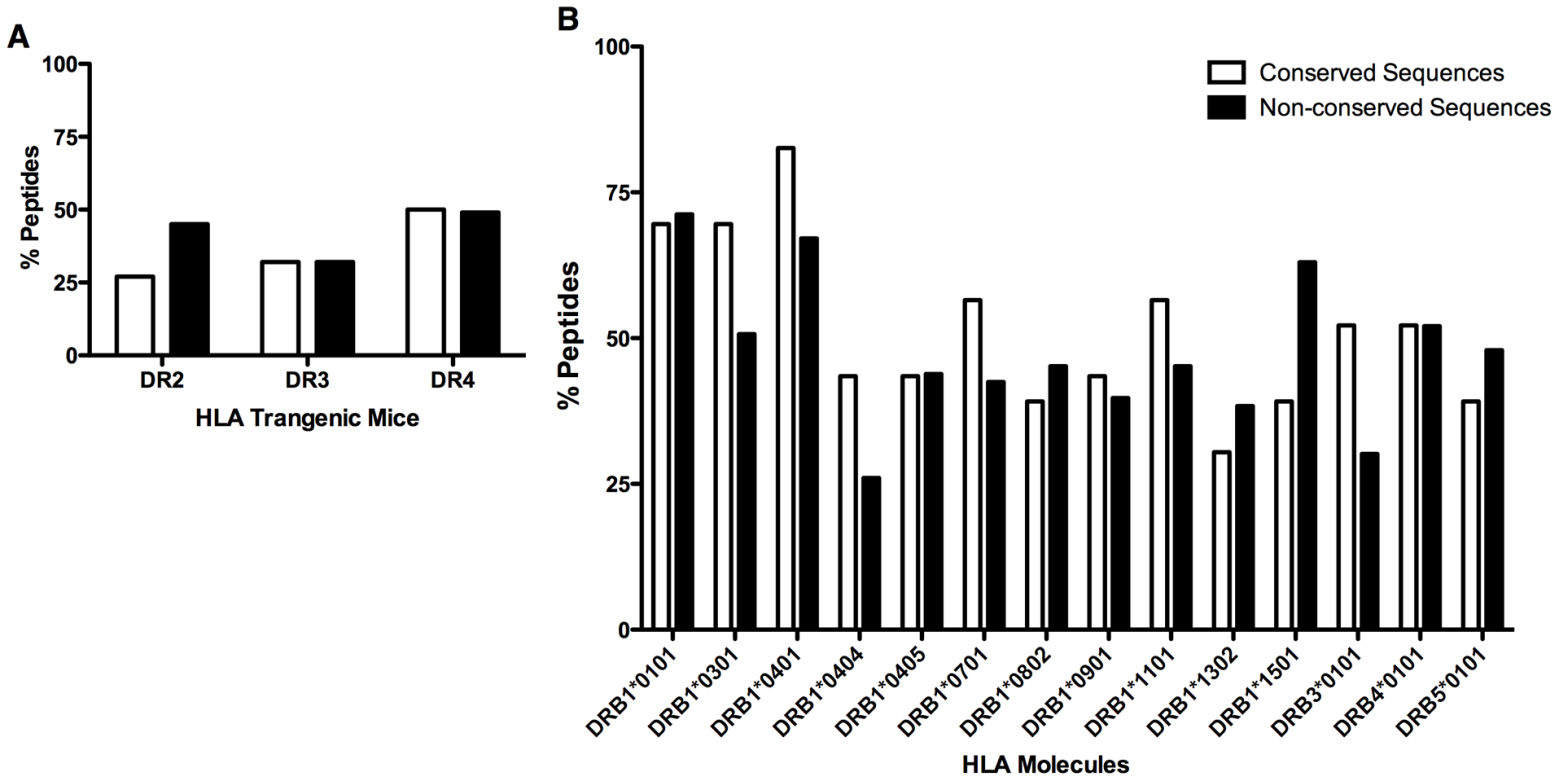
Immunogenicity and binding affinity capability of peptides containing non-conserved and pan-dengue conserved sequences. The percentage of peptides containing non-conserved and pan-dengue conserved sequences was calculated based on their ability to (A) induce T-cell responses in HLA class II transgenic mice (DR2, DR3 and DR4); or (B) to bind to 14 different HLA molecules.

A vaccine should provide broad HLA coverage for relevance at the population level. Promiscuous epitopes recognize multiple HLA molecules and are, thus, candidate epitopes for vaccine design consideration [66]. Additionally, a vaccine should also target conserved epitopes for broad coverage of viral variants. Therefore, theoretically an effective dengue vaccine would include promiscuous HLA epitopes that are highly conserved in each of the dengue serotypes. Previously, we reported several immunogenic, pan-dengue conserved sequences by use of HLA class II TgM [45]. We provide in Table 3 the list of HLA class II TgM epitopes that had HLA affinity confirmed by binding assay and contain at least nine consecutive amino acids that are pan-dengue conserved sequences. We then compared the ability of these epitopes to induce T-cell responses in TgM and to bind to multiple HLAs (among the 14 HLA molecules analyzed). The results show that peptides containing highly conserved regions are equally immunogenic (Figure 7A) and can bind to as many HLAs (Figure 7B) as compared to peptides with non-conserved sequences. Additionally, we investigated if these immunogenic conserved dengue sequences were present in the 9-mer core regions encompassing the major pockets in the groove of HLA-DR2, -DR3 and -DR4 molecules. *In silico* analysis was carried out using SMM, NN and Sturniolo prediction algorithms and the core regions were annotated and depicted in Supplementary Table S6. For most of the peptides analyzed, the three prediction methods consistently showed that pan-dengue conserved sequences directly interact (either partially or entirely) with anchor motifs in the HLA molecules, suggesting that these sequences indeed are important for HLA specificity and affinity and, thus, are suitable for epitope-based vaccine development.

**Table 3:** List of the most conserved DENV3 peptides for which immunogenicity in HLA-DR02, -DR03 and -DR04 transgenic mice was supported by binding affinity analysis to the respective HLA.

Peptide	Sequence	Transgenic mice			HLA class II binders
		DR2	DR3	DR4	
NS1_225–239_	WPKSHTLWSNGVLES	**-**	129+3	**-**	DRB1*0301, DRB1*0401, DRB1*1302, DRB3*0101
NS1_229–243_	HTLWSNGVLESDMII	**-**	205+12	214+15	DRB1*0301, DRB1*0401, DRB3*0101
NS1_261–275_	HTQTAGPWHLGKLEL	**-**	337+0	**-**	DRB1*0301, DRB4*0101
NS1_293–307_	TRGPSLRTTTVSGKL	**-**	**-**	87+41	DRB1*0301, DRB1*0401, DRB1*0701, DRB4*0101
NS3_185–199_	KKRNLTIMDLHPGSG	**-**	**-**	39+13	DRB1*0101, DR, B1*0301, DRB1*0401, DRB1*0405, DRB1*0802, DRB1*1101, DRB4*0101, DRB5*0101
NS3_293–307_	ASIAARGYISTRVGM	27+4	**-**	-	DRB1*0101, DRB1*0401, DRB1*0405, DRB1*0701, DRB1*0901, DRB1*1101, DRB1*1302, DRB1*1501, DRB5*0101
NS3_297–311_	ARGYISTRVGMGEAA	61+6	**-**	-	DRB1*0101, DRB1*0401, DRB1*0404, DRB1*0405, DRB1*0701, DRB1*1101, DRB1*1501
NS3_309–323_	EAAAIFMTATPPGTA	**-**	**-**	354+40	DRB1*0101, DRB1*0301, DRB1*0401, DRB1*0404, DRB1*0405, DRB1*0701, DRB1*0802, DRB1*0901, DRB1*1101, DRB1*1501, DRB4*0101
NS3_313–327_	IFMTATPPGTADAFP	**-**	**-**	46+11	DRB1*0101, DRB1*0401, DRB1*0701, DRB1*0802, DRB4*0101
NS3_357–371_	GKTVWFVPSIKAGND	406+130	**-**	**-**	DRB1*0101, DRB1*0301, DRB1*0401, DRB1*0404, DRB1*0405, DRB1*0701, DRB1*0802, DRB1*0901, DRB1*1101, DRB1*1302, DRB1*1501, DRB3*0101, DRB4*0101, DRB5*0101
NS3_381–395_	KKVIQLSRKTFDTEY	**-**	23+1	**-**	DRB1*0101, DRB1*0301, DRB1*0901, DRB1*1101, DRB3*0101, DRB4*0101
NS3_405–419_	FVVTTDISEMGANFK	**-**	**-**	482+27	DRB1*0301, DRB1*0401, DRB1*0404, DRB1*0405, DRB1*0802, DRB5*0101
NS3_409–423_	TDISEMGANFKADRV	**-**	114+17	**-**	DRB1*0301, DRB3*0101, DRB5*0101
NS5_295–311_	DENPYKTWAYHGSYEVK	126+10	**-**	**-**	DRB1*0101, DRB1*0301, DRB1*0401, DRB1*0404, DRB1*0405, DRB1*0701, DRB1*0901, DRB1*1501, DRB3*0101
NS5_301–316_	TWAYHGSYEVKATGSA	161+20	**-**	19+0	DRB1*0101, DRB1*0401, DRB1*0404, DRB1*0701, DRB1*0901, DRB1*1101, DRB1*1501, DRB3*0101
NS5_336–352_	MVTQMAMTDTTPFGQQR	**-**	**-**	28+0	DRB1*0101, DRB1*0301, DRB1*0401, DRB1*0404, DRB1*0405, DRB1*0701, DRB1*0802, DRB1*0901, DRB1*1302, DRB3*0101, DRB4*0101
NS5_447–463_	GSCVYNMMGKREKKLGE	**-**	**-**	16+5	DRB1*0101, DRB1*0401, DRB1*0404, DRB1*0802, DRB1*1101, DRB5*0101
NS5_501–517_	NSYSGVEGEGLHKLGYI	**-**	**-**	50+10	DRB1*0101, DRB1*0401, DRB1*0701, DRB1*0802, DRB1*0901
NS5_523–539_	KIPGGAMYADDTAGWDT	**-**	**-**	44+15	DRB1*0101, DRB1*0301, DRB1*0401, DRB1*1101, DRB3*0101, DRB4*0101
NS5_564–580_	ANAIFKLTYQNKVVKVQ	849+8	**-**	**-**	DRB1*0101, DRB1*0301, DRB1*0401, DRB1*0701, DRB1*0901, DRB1*1101, DRB1*1302, DRB1*1501, DRB3*0101, DRB4*0101, DRB5*0101
NS5_588–604_	VMDIISRKDQRGSGQVG	**-**	87+7	**-**	DRB1*0301, DRB1*1101
NS5_652–668_	VERLKRMAISGDDCVVK	**-**	255+22	**-**	DRB1*0101, DRB1*0301, DRB1*0401, DRB1*0404, DRB1*0405, DRB1*0701, DRB1*1101, DRB1*1302, DRB1*1501, DRB3*0101, DRB4*0101, DRB5*0101
NS5_765–779_	MYFHRRDLRLASNAI	75+16	**-**	531+10	DRB1*0101, DRB1*0401, DRB1*0404, DRB1*0405, DRB1*0701, DRB1*0802, DRB1*0901, DRB1*1101, DRB1*1302, DRB1*1501, DRB3*0101, DRB4*0101, DRB5*0101

A representative ELISPOT data is shown along with the minimum peptide concentration that activated memory T-cells (functional avidity) is included. In addition, IC50 (nM) for HLA-DR02, -DR03 and -DR04 as well as the number of additional potential HLA binders (as many as 11) are also shown. Binding affinity is characterized by IC50 below 1000 nM (in bold).

Pan-dengue conserved sequences are shown underlined.

## Discussion

CD4 and CD8 T-cells have been shown to mediate protection against lethal dengue virus challenges in a mice model [10], [11]. However, limited dengue-specific T-cell epitopes have been reported, specifically for DENV3, a serotype responsible for several outbreaks worldwide [31], [32], [33], [34], [35], [36], [37], [38]. Herein, we have performed an extensive T-cell epitope mapping and identified more than 90 novel potential T-cell epitopes for Env, NS1, NS3 and NS5 proteins of DENV3, by use of TgM expressing a set of HLA class I and II molecules highly frequent in the Caucasian population.

Herein we identified 185 immunogenic peptides from the entire peptide library analyzed, 13 of which were HLA class I-restricted epitopes, whereas 172 were HLA class II-restricted epitopes. The number of epitopes found on HLA class II TgM outnumbered the HLA class I counterpart, which is consistent with other studies using the same TgM animal model and similar immunization strategies [53], [67]. The combination of immunization strategy (with peptide pool), the difference in topology between HLA class I and II molecules and the HLA class I epitope processing mechanism [68] might explain the reasons for the paucity of HLA class I epitopes identified in our study and, thus, revealing to be a caveat for identification of HLA class I restricted epitopes.

**Figure 8:**
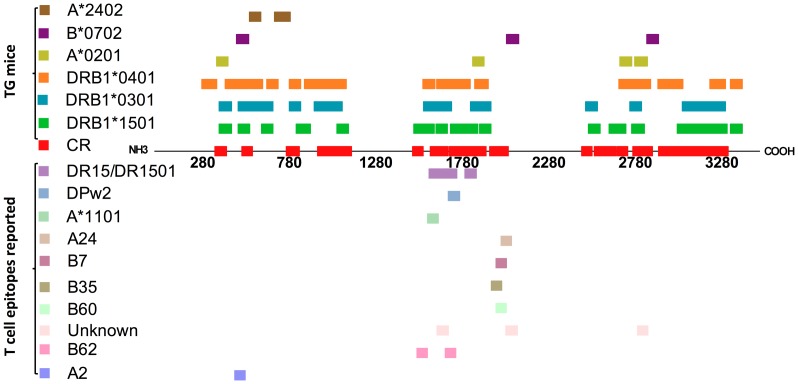
DENV3 polyprotein schema showing the localization of potential T-cell epitopes identified in this study as well as the pan-dengue conserved regions (CR) and the list of DENV3 T-cell epitopes reported in the literature.

Binding assay analysis was done in order to confirm the HLA affinity suggested by TgM immunogenicity data. HLA affinity correspondence between the two data was observed for 9 HLA class I and 97 HLA class II epitopes. A schema of DENV3 proteome with the location of each confirmed epitope as well as those reported in the literature are illustrated in Figure 8. A number of these epitopes were shown to be quite promiscuous, binding to as many as 14 HLA class II molecules. Besides HLA promiscuity improving vaccine population coverage, multiple HLA binding motifs within a peptide antigen have been shown to enhance T-cell immunogenicity in humans vaccinated with attenuated yellow fever 17DD vaccine [69].

A comparison between DR2, DR3, and DR4 TgM immunogenicity and binding affinity data reported herein showed that not all the peptides with binding affinity to the HLA(s) induced an immune response in the transgenic mice. This is likely related to how T-cells are activated. In order to trigger T-cell responses there are two essential steps: (1) the epitope must bind to the HLA molecule; and (2) the T-cell receptor (TCR) on T-cells must recognize the HLA-epitope complex. Binding affinity only takes into consideration the step 1, while immunogenicity is more complex, taking into account both steps. Anything that influences epitope presentation (e.g. epitope dominance) will affect the T-cell activation and, consequently, will compromise the step 2. Thus, discrepancies observed between binding affinity and immunogenicity were most likely due to the fact that some peptides failed to activate T-cells possibly due to epitope competition and/or dominance during animal immunization.

We selected a subset of epitopes that HLA affinity and TgM immunogenicity were corresponding for assessment of their *ex vivo* immunogenicity (ability to activate memory T-cells) in individuals with matched HLA and history of DENV3 infections. These individuals were enrolled in a dengue cohort established in Recife, Brazil, and described elsewhere [37]. The peptides NS3_399–407_ and NS5_318–326_ (A02 epitopes); NS5_389–398_, Env_226–234_ and NS3_593–601_ (B07 epitopes); Env_126–140_, Env_231–245_, NS1_69–83_, NS1_85–99_ and NS3_357–371_ (DR2 epitopes) activated memory T-cells of at least one of the subjects analyzed, suggesting that these epitopes are naturally processed and presented to T-cells during the course of dengue infection and, thus, are relevant to humans. However, we cannot rule out the possibility that the epitopes tested could have been presented by HLA molecules other than those studied herein or the memory T-cell clones activated were only cross-reactive to DENV3. Further studies are needed to determine the HLA restriction and the specificity of these T-cell clones.

We also analyzed the ability of the epitopes to prime naïve T-cells. Two selected DENV3 epitopes, NS3_399–407_ (and its variants on DENV2 and DENV4 serotypes) and NS5_389–398_, were analyzed for their ability to activate naïve T-cells from subjects either HLA-A02 or HLA-B07 positives who had never been exposed to dengue virus infection by any serotype (dengue IgG negative). Naïve T-cells were successfully primed by both DENV3 epitopes (NS3_399–407_ and NS5_389–398_) and the NS3_399–407_ variant in DENV4, but not by the DENV2 variant. The residues lysine (K) and leucine (L) at the amino termini of the peptide that are part of the HLA-A*0201 binding motif (www.syfpeithi.de) seemed to determine the immunogenicity of the peptides as both residues were present in the DENV3 NS3 epitope and its DENV4 variant, but not in the DENV2 variant (Table 2). Notably, DENV3 NS3_399–407_ -primed CD8 T-cells have been shown to recognize both DENV2 and DENV4 variants leading to a dysfunctional T-cell response [70]. Thus, more studies are needed to determine the role of these altered peptide ligands on disease outcome. Nonetheless, the data suggests that the epitopes are naturally processed and can potentially prime naïve T-cells, which is important for vaccine design.

Our epitope screening strategy revealed HLA class I epitopes found only in non-conserved regions of the virus proteome. We analyzed the intra- and inter-serotype variants of the A02-restricted epitope NS3_399–407_ (KLNDWDFVV) among the sequences deposited in the NCBI Entrez Protein Database [45] to assess for the possibility of altered peptide ligands within DENV3 and between the serotypes. Based on the analysis, an intra-serotype variant ( RLNDWDFVV) and four inter-serotype variants were identified (Table 2). *In silico* analysis showed that all, but DENV1 variant had HLA-A*0201 binding motif, and, thus, capable of potentially acting as an altered peptide ligand, leading to aberrant memory T-cell responses.

A study by Hertz et al [71] using targeting efficiency analysis of different HLA class I molecules has shown that HLA molecules preferentially target conserved regions on the proteome of different viruses [71]. This was thought to be the case on DENV as well [12]. Nevertheless, virus belonging to *Flaviviridae* family, such as DENV, are exception to this rule, since there is an increased preference of HLA molecules to bind non-conserved regions among the different virus serotypes [71]. However, the authors found an association between preferential HLA targeting to conserved regions and protection against dengue severity caused by DENV2 [71]. Hence, it seems that targeting conserved sequences is desirable not only in terms of achieving a broad and efficient pan-dengue immune responses, but also to protect against disease severity. In contrast to the HLA class I, where we did not find conserved immunogenic regions, we identified 23 HLA class II immunogenic peptides that contained sequences highly conserved in each of the four dengue serotypes (pan-dengue conserved sequences) [45]. In TgM model, these conserved peptides were as immunogenic (among DR2, DR3 and DR4) and bound to as many HLA molecules as the peptides containing non-conserved sequences. Notably, these sequences are predicted to actively interact with the major pockets in the groove of HLA-DR2, -DR3 and -DR4 molecules, thus, defining the importance of these pan-dengue conserved sequences for the affinity and specificity of the epitopes identified. Moreover, some of the epitopes bearing pan-dengue conserved sequences could bind as many as 14 different HLA molecules, suggesting that these immunogenic conserved sequences could trigger T-cell responses in a vast number of people. However, it is important to highlight that these immunogenic and HLA promiscuous peptides are not optimum epitopes, thus, additional analysis are needed in order to identify the exact sequences responsible for HLA affinity and ultimately T-cell activation. The importance of these epitopes in preventing dengue infection and disease severity as well as population coverage also needs to be further addressed.

Currently there are as many as 15 reported human T-cell epitopes for DENV3 in the literature (Table 4; Figure 8; as of December 2012). Among these, the DR2-restricted epitope NS3_348–362_ (GNEWITDFVGKTVWF) reported by Mangada & Rothman [25] using a cohort of individuals immunized against DENV3 virus correlated with four overlapping DENV3 DR2 TgM epitopes identified herein, covering a larger region (NS3_345–370_). Two of the four overlapping epitopes (NS3_349–363_ and NS3_357–370_) had the specificity for DR2 molecule confirmed by binding assay. Thus, there are possible multiple overlapping epitope in the region described by Mangada & Rothman. Notably, the peptide NS3_357–370_ (GKTVWFVPSIKAGND), which overlapped 6 amino acids with the epitope reported by Mangada & Rothman [25], was observed to bind to DR2 with even greater affinity and elicit memory T-cell response at a lower concentration. Additionally, we confirmed the HLA affinity of some of the previously reported epitopes. For instance, we identified a potential DR2 (EEMFKK**RNLTIMDLH** ) and a DR4 (KK**RNLTIMDLHPGSG** ) epitope overlapping the NS3_187–201_ (RNLTIMDLHPGSGKT) with no reported HLA affinity; similarly, a B07 (RPRWLDA) epitope within the NS3_585–599_ (KEGEKKKLRPRWLDA) [25]. Recently, Weiskopf et al [17] reported DENV2 T-cell epitopes using IFN α/βR KO HLA transgenic mice, an animal model that supports dengue replication and also expresses HLA-A*0201, HLA-B*0702, among other HLAs. In their study, the authors used DENV2 predicted epitopes for splenocyte challenge and a mouse-adapted DENV2 strain for immunization, and among the epitopes identified were five with identity greater than 89% in DENV3 (identity not observed for other serotypes). Among these were three HLA-B*0702 epitopes (NS3_205–213_: LPAIVREAI; NS3_223–232_: APTRVVAAEM; NS3_276–283_: VPNYNLIIM), however, none was positive in our study with DENV3 involving TgM. This could be because of the amino acid differences between the serotypes, however the increased virus replication within the animal model used might have contributed to the increased breadth and magnitude of T-cell response. Additionally, other factors might concomitantly facilitate the T-cell responses in the IFN α/βR KO HLA transgenic mice, such as increased antigen availability.

**Table 4:** List of DENV3 epitopes along with HLA affinity (if known) described in the literature and comparison with the immunogenicity and binding affinity of the peptides tested in this study.

Peptide Tested			DENV3 Epitopes Reported	
Position	Sequence	TGM/Binding assay	Protein (Sequence)/HLA affinity	References
Env_206–220_Env_211–225_	VHRQW**FFDLPLPWT**S **FFDLPLPWT**SGATTE	Negative/NDNegative/ND	Env_211–219_ (FFDLPLPWT)/A*2	[20]
NS3_129–143_NS3_133–147_	DFKP**GTSGSPIINRE** **GTSGSPIINRE**GKVV	Negative/NDNegative/ND	NS3_133–142_ (GTSGSPIINR;GTSGSPIINRE)/A*11, A*1101	[15], [21], [27]
NS3_137–151_NS3_141–155_NS3_145–159_	SPII**NREGKVVGLYG** **NREGKVVGLYGNGVV** **KVVGLYGNGVV**TKNG	DR3/DR2,DR3Negative/NDNegative/ND	NS3_141–155_ (NREGKVVGLYGNGVV)/DRB1*1501	[25]
NS3_181–195_NS3_185–199_NS3_189–203_NS3_193–207_	EEMFKK**RNLTIMDLH** KK**RNLTIMDLHPGSG** **LTIMDLHPGSGKT**RK **DLHPGSGKT**RKYLPA	DR2/DR2DR4/DR4Negative/NDNegative/ND	NS3_187–201_ (RNLTIMDLHPGSGKT)	[25]
NS3_197–211_NS3_201–215_	GSGKT**RKYLPAIVRE** T**RKYLPAIVRE**AIKR	Negative/NDDR3, DR4/DR4	NS3_202–211_ (RKYLPAIVRE)/DRB1*15	[28]
NS3_221–235_NS3_225–239_NS3_229–243_	ILA**PTRVVAAEMEEA** **TRVVAAEMEEALKG**L **AAEMEEALKG**LPIRY	Negative/NDNegative/NDNegative/ND	NS3_224–238_ (PTRVVAAEMEEAMKG; TRVVAAEMEEA)/DRB1*15; DRB1*1501	[24], [25]
NS3_237–251_NS3_241–255_	KGLP**IRYQTTATK**SE **IRYQTTATK**SEHTGR	DR4/DR2, DR4DR4/DR4	NS3_241–249_ (IRYQTTATK)/DRB1*15	[28]
NS3_249–263_NS3_253–267_NS3_257–271_	KSE**HTGREIVDLMCH** **TGREIVDLMCHATF**T **IVDLMCHATFT**MRLL	Negative/NDNegative/NDNegative/ND	NS3_254–266_ (HTGREIVDLMCHAT**E** ; EIVDLMCHAT;VDLMCHATFT)/DPw2	[23], [25], [26]
NS3_345–359_NS3_349–363_NS3_353–367_NS3_357–370_	WNS**GNEWITDFAGKT** **NEWITDFAGKTVWF**V **TDFAGKTVWF**VPSIK **GKTVWF**VPSIKAGND	DR2*/NegativeDR2/DR2DR2*/NegativeDR2/DR2	NS3_348–362_ (GNEWITDFVGKTVWF)/DRB1*1501	[25]
NS3_497–511_NS3_501–515_	DNIN**TPEGIIPAL**FE **TPEGIIPAL**FEPERE	Negative/NDNegative/ND	NS3_501–509_ (TPEGIIPAL)/B*35	[29], [74]
NS3_521–535_NS3_525–539_NS3_529–543_	DGEYRLK**GESRKTFV** RLK**GESRKTFVEL**MR **ESRKTFVEL**MRRGDL	Negative/NDNegative/NDNegative/ND	NS3_528–535_ (GESRKTFVE)/B*07; NS3_528–536_ (GESRKTFVEL)/B*60	[18], [27]
NS3_553–547_NS3_557–571_	SEGI**KYTDRKWCF**DG **KYTDRKWCF**DGERNN	Negative/NDNegative/ND	NS3_557–565_ (KYTDRKWCF)/A*24	[16]
NS3_577–591_NS3_581–595_NS3_585–599_NS3_589–603_NS3_593–607_	NMDVEIWT**KEGEKKK** EIWT**KEGEKKKLRPR** **KEGEKKKLRPRWLDA** **KKKLRPRWLDA** RTYS **RPRWLDA** RTYSDPLA	Negative/NDNegative/NDNegative/NDB07/B07B07/B07	NS3_585–599_ (KEGEKKKLRPRWLDA)	[25]
NS5_318–334_NS5_324–340_NS5_330–346_	SMINGVVKLLT**KPWDVV** VKLLT**KPWDVVPMV**TQM **PWDVVPMV**TQMAMTDTT	A02/A02Negative/NDDR4/DR4	NS5_329–337_ (KPWDVVPTV)	[22]

The sequences in bold represent the epitopes previously identified within the peptides used in this study. ND: non-determined.

The use of HLA TgM has been shown to be useful tool for the identification of potential T-cell epitopes, although a validation step is required [44]. Several studies have shown correlation between epitopes identified in HLA TgM and humans [42], [43], [53]. Herein, we have identified a vast repertoire of potential DENV3 T-cell epitopes by use of TgM, with HLA affinity validated for many by use of binding assay, and immunogenicity confirmed for a subset in HLA-matched subjects with history of dengue infection. The analysis of intra- and inter-serotype variants performed is important to identify and understand the role of altered peptide ligands and T-cell activation in the context of disease severity caused by DENV and on the other hand population coverage. Notably, a more extensive human validation study is currently underway employing the same peptide library used herein. The data to date reveal that twenty-seven (27) of the TgM immunogenic peptides (considering only Envelope, NS1 and NS3; data not shown) are also immune-prevalent (recognized by at least 10% of the subjects tested) in a human cohort of subjects naturally exposed to DENV3 (Souza *et al.*, manuscript in preparation).

The most advanced dengue vaccine candidates in clinical trials include the Walter Reed Army Institute of Research-GlaxoSmithKline (WRAIR-GSK) new live attenuated virus vaccine that are in phase II [72] and the live chimeric virus vaccines, from Sanofi Pasteur (ChimeriVax), currently in phase III [73]. The WRAIR-GSK vaccine can induce immune responses against dengue structural and non-structural viral proteins, while ChimeriVax induces responses to dengue prM and Env proteins and yellow fever non-structural proteins. The ability of these vaccines to induce T-cell responses has been reported [74], [75], [76]. The complete T-cell repertoires of these vaccines are not known, but they can potentially induce T-cell responses to both conserved and non-conserved epitopes. Herein we report highly conserved, HLA promiscuous dengue T-cell epitopes (mostly found in the non-structural proteins of the virus) that are capable of inducing T-cell responses against the majority of dengue variants of each of the four serotypes and applicable to human population. We postulate that it would be possible to engineer a dengue vaccine that could target specifically the conserved T-cell epitopes. This may be addressed by priming the immune system with highly conserved, HLA promiscuous T-cell epitopes concomitantly with dengue prM/Env proteins. This may not only help to produce high affinity neutralizing antibodies, but also would expand T-cell clones specific for highly conserved dengue T-cell epitopes. These T-cells would be effectively activated after virus exposure in the field to produce cytokines (e.g. IFN-γ) associated with disease protection. Further studies are needed to identify the cytokine repertoire that such epitopes may induce and understand their role in dengue protection/pathogenesis, as well as establish proper epitope delivery for efficient antigen presentation and T-cell activation. The data reported herein are valuable resource for further studies investigating the role of T-cells in dengue protection/immunopathogenesis and design of tetravalent dengue vaccine.

## Supporting Information

Material S1 Full repertoire of DENV3 T-cell epitopes identified in HLA transgenic mice with their binding affinity data.
(XLSX)

Table S1 Demographic and serology data for the volunteers used in the immunogenicity studies.
(DOC)

Table S2 HLA typing data for the volunteers used in the immunogenicity studies.
(DOC)

Table S3 Overview of the peptide libraries analyzed.
(DOC)

Table S4 HLA affinity profile of overlapping peptides covering the entire sequence of DENV3 Envelope, NS1, NS3 and NS5 based on the immunogenicity assay in HLA transgenic mice.
(DOC)

Table S5 Comparative analysis of immunogenicity and the binding affinity properties for the peptides positive in HLA-DR2, -DR3 and -DR4 transgenic mice.
(DOC)

Table S6 Determination of core motifs of HLA class II epitopes containing pan-dengue conserved sequences.
(DOC)
