# Supplementary material for: Identification of Conserved and HLA Promiscuous DENV3 T-Cell Epitopes
Source: PLoS Negl Trop Dis. 2013 Oct 10;7(10):e2497. doi: 10.1371/journal.pntd.0002497 (PMC3794980; doi:10.1371/journal.pntd.0002497)
Supplement: Table S1 — Demographic and serology data for the volunteers used in the immunogenicity studies. (DOC) [file pntd.0002497.s002.doc]

TABLE S1. Demographic and serology data for the volunteers used in the immunogenicity studies.

| **Patient ID** | **Age** | **Sex** | **Early phase serum sample** | | | | |  | **Late phase serum sample** | | | | |
| --- | --- | --- | --- | --- | --- | --- | --- | --- | --- | --- | --- | --- | --- |
|  |  |  | **Days*** | **PRNT Titers** | | | |  | **Days*** | **PRNT Titers** | | | |
|  |  |  |  | **DENV1** | **DENV2** | **DENV3** | **DENV4** |  |  | **DENV1** | **DENV2** | **DENV3** | **DENV4** |
| 764 | 9 | F | 9 | <1:20 | <1:20 | 1:80 | <1:20 |  | 17 | <1:20 | 1:20 | 1:320 | <1:20 |
| 775 | 12 | M | 7 | 1:40 | 1:320 | 1:1280 | <1:20 |  | 24 | 1:320 | 1:320 | 1:1280 | <1:20 |
| 799 | 12 | M | 6 | 1:20 | 1:20 | 1:80 | <1:20 |  | 35 | 1:20 | 1:20 | 1:1280 | <1:20 |
| 850 | 11 | M | 11 | 1:80 | 1:640 | 1:1280 | <1:20 |  | 39 | 1:80 | 1:640 | ≥1:1280 | <1:20 |
| 856 | 13 | F | 2 | 1:160 | 1:20 | 1:320 | <1:20 |  | 128 | 1:160 | 1:640 | 1:640 | <1:20 |

* Days after onset of symptoms when the volunteers were first recruited to the cohort.
