# Supplementary material for: Identification of Conserved and HLA Promiscuous DENV3 T-Cell Epitopes
Source: PLoS Negl Trop Dis. 2013 Oct 10;7(10):e2497. doi: 10.1371/journal.pntd.0002497 (PMC3794980; doi:10.1371/journal.pntd.0002497)
Supplement: Table S2 — HLA typing data for the volunteers used in the immunogenicity studies. (DOC) [file pntd.0002497.s003.doc]

Table S2. HLA typing data for the volunteers used in the immunogenicity studies.

| **Patient ID** | **HLA Type** | **Figure Reference** |
| --- | --- | --- |
| 764 | A(**02**,68); B(45,51); Cw(16,15); DRB1 (10,13); DQ (05,06) | Fig. 3A |
| 775 | A(23,66); B(44,58); Cw(04,06); DRB1 (07,**15**); DQ (02,06) | Fig. 4C |
| 799 | A(**02**,68); B(51,ND); Cw(04,16); DRB1 (13,13); DQ (03,05) | Fig. 3B |
| 850 | A(01,29); B(**07**,44); Cw(07,16); DRB1 (07,**15**); DQ (02,06) | Fig. 3C; Fig. 4A |
| 856 | A(03,24); B(**07**,18); Cw(05,07); DRB1 (05,**15**); DQ (02,06) | Fig. 3D; Fig. 4B |

ND – Not determined
