# Supplementary material for: Identification of Conserved and HLA Promiscuous DENV3 T-Cell Epitopes
Source: PLoS Negl Trop Dis. 2013 Oct 10;7(10):e2497. doi: 10.1371/journal.pntd.0002497 (PMC3794980; doi:10.1371/journal.pntd.0002497)
Supplement: Table S3 — Overview of the peptide libraries analyzed. (DOC) [file pntd.0002497.s004.doc]

TABLE S3. Overview of the peptide libraries analyzed.

| **Protein** | **Length (aa)** | **Total overlapping peptides** | **Peptides containing pan dengue conserved sequences (%)** |
| --- | --- | --- | --- |
| Envelope | 493 | 95 | 5 (5) |
| NS1 | 327 | 76 | 14 (19) |
| NS3 | 619 | 150 | 23 (15) |
| NS5 | 900 | 156 | 36 (23) |
