# Supplementary material for: Identification of Conserved and HLA Promiscuous DENV3 T-Cell Epitopes
Source: PLoS Negl Trop Dis. 2013 Oct 10;7(10):e2497. doi: 10.1371/journal.pntd.0002497 (PMC3794980; doi:10.1371/journal.pntd.0002497)
Supplement: Table S4 — HLA affinity profile of overlapping peptides covering the entire sequence of DENV3 Envelope, NS1, NS3 and NS5 based on the immunogenicity assay in HLA transgenic mice. (DOC) [file pntd.0002497.s005.doc]

TABLE S4. HLA affinity profile of overlapping peptides covering the entire sequence of DENV3 Envelope, NS1, NS3 and NS5 based on the immunogenicity assay in HLA transgenic mice.

| **Protein** | **HLA class I positive peptides** | **HLA class II positive peptides** | **Total immunogenic peptides (%)** | **HLA binding** | | | | | |
| --- | --- | --- | --- | --- | --- | --- | --- | --- | --- |
|  |  |  |  | **HLA Class I** | | | **HLA class II** | | |
|  |  |  |  | **A2** | **A24** | **B7** | **DR2** | **DR3** | **DR4** |
| Envelope | 5 | 36 | 41 (43) | 1 | 3 | 1 | 10 | 12 | 14 |
| NS1 | 0 | 31 | 31 (41) | 0 | 0 | 0 | 5 | 12 | 14 |
| NS3 | 3 | 48 | 51 (34) | 1 | 0 | 2 | 20 | 17 | 11 |
| NS5 | 4 | 58 | 62 (40) | 3 | 0 | 1 | 29 | 9 | 20 |
