# Supplementary material for: Identification of Conserved and HLA Promiscuous DENV3 T-Cell Epitopes
Source: PLoS Negl Trop Dis. 2013 Oct 10;7(10):e2497. doi: 10.1371/journal.pntd.0002497 (PMC3794980; doi:10.1371/journal.pntd.0002497)
Supplement: Table S5 — Comparative analysis of immunogenicity and the binding affinity properties for the peptides positive in HLA-DR2, -DR3 and -DR4 transgenic mice. (DOC) [file pntd.0002497.s006.doc]

TABLE S5. Comparative analysis of immunogenicity and the binding affinity properties for the peptides positive in HLA-DR2, -DR3 and -DR4 transgenic mice.

| **Peptides** | **HLA** | | |
| --- | --- | --- | --- |
|  | **DR2** | **DR3** | **DR4** |
| Positive in TG mice | 60 | 49 | 56 |
| Affinity confirmed  (% of positive peptides) | 39  (65%) | 26  (53%) | 47  (84%) |
