# Supplementary material for: Identification of Conserved and HLA Promiscuous DENV3 T-Cell Epitopes
Source: PLoS Negl Trop Dis. 2013 Oct 10;7(10):e2497. doi: 10.1371/journal.pntd.0002497 (PMC3794980; doi:10.1371/journal.pntd.0002497)
Supplement: Table S6 — Determination of core motifs of HLA class II epitopes containing pan-dengue conserved sequences. (DOC) [file pntd.0002497.s007.doc]

TABLE S6. Determination of core motifs of HLA class II epitopes containing pan dengue conserved sequences.

| **Peptide** | **Sequence** | **Prediction Method** | **Predicted Core Sequences** | | |
| --- | --- | --- | --- | --- | --- |
|  |  |  | **DR2** | **DR3** | **DR4** |
| NS1_225-239_ | WPKSHTLWSNGVLES | SMM | - | TLWSNGVLE | - |
|  |  | NN | - | LWSNGVLES | - |
|  |  | Sturniolo | - | LWSNGVLES | - |
| NS1_229-243_ | HTLWSNGVLESDMII | SMM | - | LWSNGVLES | LWSNGVLES |
|  |  | NN | - | LWSNGVLES | TLWSNGVLE |
|  |  | Sturniolo | - | LWSNGVLES | LWSNGVLES |
| NS1_261-275_ | HTQTAGPWHLGKLEL | SMM | - | TQTAGPWHL | - |
|  |  | NN | - | TQTAGPWHL | - |
|  |  | Sturniolo | - | HTQTAGPWH | - |
| NS1_293-307_ | TRGPSLRTTTVSGKL | SMM | - | - | LRTTTVSGK |
|  |  | NN | - | - | SLRTTTVSG |
|  |  | Sturniolo | - | - | LRTTTVSGK |
| NS3_185-199_ | KKRNLTIMDLHPGSG | SMM | - | - | LTIMDLHPG |
|  |  | NN | - | - | TIMDLHPGS |
|  |  | Sturniolo | - | - | LTIMDLHPG |
| NS3_293-307_ | ASIAARGYISTRVGM | SMM | ARGYISTRV | - | - |
|  |  | NN | ARGYISTRV | - | - |
|  |  | Sturniolo | IAARGYIST | - | - |
| NS3_297-311_ | ARGYISTRVGMGEAA | SMM | ARGYISTRV | - | - |
|  |  | NN | ARGYISTRV | - | - |
|  |  | Sturniolo | ISTRVGMGE | - | - |
| NS3_309-323_ | EAAAIFMTATPPGTA | SMM | - | - | FMTATPPGT |
|  |  | NN | - | - | IFMTATPPG |
|  |  | Sturniolo | - | - | IFMTATPPG |
| NS3_313-327_ | IFMTATPPGTADAFP | SMM | - | - | FMTATPPGT |
|  |  | NN | - | - | FMTATPPGT |
|  |  | Sturniolo | - | - | IFMTATPPG |
| NS3_357-371_ | GKTVWFVPSIKAGND | SMM | VWFVPSIKA | - | - |
|  |  | NN | VWFVPSIKA | - | - |
|  |  | Sturniolo | VWFVPSIKA | - | - |
| NS3_381-395_ | KKVIQLSRKTFDTEY | SMM | - | IQLSRKTFD | - |
|  |  | NN | - | IQLSRKTFD | - |
|  |  | Sturniolo | - | IQLSRKTFD | - |
| NS3_405-419_ | FVVTTDISEMGANFK | SMM | - | - | VTTDISEMG |
|  |  | NN | - | - | FVVTTDISE |
|  |  | Sturniolo | - | - | VVTTDISEM |
| NS3_409-423_ | TDISEMGANFKADRV | SMM | - | MGANFKADR | - |
|  |  | NN | - | MGANFKADR | - |
|  |  | Sturniolo | - | MGANFKADR | - |
| NS5_295-311_ | DENPYKTWAYHGSYEVK | SMM | PYKTWAYHG | - | - |
|  |  | NN | TWAYHGSYE | - | - |
|  |  | Sturniolo | WAYHGSYEV | - | - |
| NS5_301-316_ | TWAYHGSYEVKATGSA | SMM | WAYHGSYEV | - | YHGSYEVKA |
|  |  | NN | TWAYHGSYE | - | YHGSYEVKA |
|  |  | Sturniolo | WAYHGSYEV | - | WAYHGSYEV |
| NS5_336-352_ | MVTQMAMTDTTPFGQQR | SMM | - | - | AMTDTTPFG |
|  |  | NN | - | - | MAMTDTTPF |
|  |  | Sturniolo | - | - | MAMTDTTPF |
| NS5_447-463_ | GSCVYNMMGKREKKLGE | SMM | - | - | YNMMGKREK |
|  |  | NN | - | - | VYNMMGKRE |
|  |  | Sturniolo | - | - | YNMMGKREK |
| NS5_501-517_ | NSYSGVEGEGLHKLGYI | SMM | - | - | YSGVEGEGL |
|  |  | NN | - | - | YSGVEGEGL |
|  |  | Sturniolo | - | - | VEGEGLHKL |
| NS5_523-539_ | KIPGGAMYADDTAGWDT | SMM | - | - | MYADDTAGW |
|  |  | NN | - | - | MYADDTAGW |
|  |  | Sturniolo | - | - | MYADDTAGW |
| NS5_564-580_ | ANAIFKLTYQNKVVKVQ | SMM | KLTYQNKVV | - | - |
|  |  | NN | FKLTYQNKV | - | - |
|  |  | Sturniolo | FKLTYQNKV | - | - |
| NS5_588-604_ | VMDIISRKDQRGSGQVG | SMM | - | DIISRKDQR | - |
|  |  | NN | - | DIISRKDQR | - |
|  |  | Sturniolo | - | MDIISRKDQ | - |
| NS5_652-668_ | VERLKRMAISGDDCVVK | SMM | - | MAISGDDCV | - |
|  |  | NN | - | ISGDDCVVK | - |
|  |  | Sturniolo | - | ISGDDCVVK | - |
| NS5_765-779_ | MYFHRRDLRLASNAI | SMM | FHRRDLRLA | - | HRRDLRLAS |
|  |  | NN | DLRLASNAI | - | YFHRRDLRL |
|  |  | Sturniolo | YFHRRDLRL | - | MYFHRRDLR |

*Underlined sequences represent pan-dengue conserved sequences [45].
